# Diagnostic accuracy of procalcitonin in maternal plasma to detect early intra-amniotic infection in preterm premature rupture of the membranes with respect of highvaginal swab as gold standard

**DOI:** 10.12669/pjms.38.1.4436

**Published:** 2022

**Authors:** Rabia Farooqui, Qurat-ul-Aman Siddiqui

**Affiliations:** 1 Dr. Rabia Farooqui, MBBS, FCPS. Senior Registrar, Department of Obstetrics & Gynaecology, Liaquat National Hospital and Medical College, Karachi, Pakistan; 2 Dr. Qurat-ul-Aman Siddiqui, MBBS, MCPS, FCPS. Associate Professor, Department of Obstetrics & Gynaecology, Australian Concept Infertility Medical Centre, Karachi, Pakistan. Liaquat National Hospital and Medical College, Karachi, Pakistan

**Keywords:** Procalcitonin, Chorioamnionitis, Preterm premature rupture of the membranes (PPROM), Intra-amniotic infection, High Vaginal Swab

## Abstract

**Objective:**

To determine the diagnostic accuracy of procalcitonin in maternal plasma to detect early intra-amniotic infection in Preterm premature rupture of the membranes (PPROM) with respect of high vaginal swab as gold standard

**Methods:**

A cross-sectional study was conducted at Liaquat National Hospital, Karachi, from February to August 2017. The blood sample of women with PPROM were collected to measure procalcitonin level. PCT1 and PCT2 were run along with the sample for the accuracy of the results. Sensitivity, specificity, PPV, NPV, and diagnostic accuracy of procalcitonin were calculated taking HVS C/S as gold standard.

**Results:**

Out of total 150 women, mean age was 28.78±4.79 years. Mean gestational age was 30.79±3.07 weeks. Mean procalcitonin level was 0.13±0.24 ng/ml. Intra-amniotic infection was diagnosed in 48.7% cases through procalcitonin levels and 51.3% through HVS culture and sensitivity. Sensitivity, Specificity, PPV (Positive predictive value), NPV (Negative predictive value) and accuracy were 87%, 91.8%, 91.78%, 87%, and 89.3% respectively. For females with gestational age ≤32 weeks, sensitivity, specificity, and diagnostic accuracy were 83.9%, 90.4%, and 87.03% respectively. For females with gestational age >32 weeks, sensitivity, specificity, and diagnostic accuracy were 95.2%, 92.5%, and 95.23% respectively.

**Conclusion:**

Diagnostic accuracy of maternal blood procalcitonin levels were found satisfactory in detection of early intra-amniotic infection in PPROM.

## INTRODUCTION

Premature rupture of membrane (PROM) is a common condition in obstetrics, linking the amniotic cavity with the bacterial flora of the cervix with the vagina. The main risk that can occur in any case of PPROM is occurrence of chorioamnionitis, thereby increasing the neonatal as well as maternal morbidity and mortality.^1^ One of the main etiological factors of PROM and Preterm-PROM (PPROM) is primary intra-amniotic infection of subclinical nature. Such cases require early identification so as to make a decision regarding proper therapeutic management. The ways of to determine this subclinical nature of infection detection are limited. Literature has been proved that measurement of inflammatory in maternal plasma may help to predict fetal compromise and neonatal morbidity that is directly related to maternal relation.^2^,^3^

According to a study, the prevalence of PPROM was 2.3% with almost 53% showing evidence of chorioamnionitis.^4^ PPROM is a major preterm birth cause, which can lead to a high level of perinatal and maternal morbidity and mortality.^5^ Perinatal morbidity includes respiratory distress syndrome, neonatal sepsis, necrotizing enterocolitis (NEC) and fetal death.^6^ For improving neonatal outcomes, appropriate evaluation and management are necessary.^7^,^8^

Initiating work to diagnose infection at an early stage is of prime importance. If antibiotic therapy is promptly initiated, its sequelae can be reduced and pronounced. There are, however, few tests to detect early infection on which to rely.^9^ Procalcitonin (PCT) has been recently acknowledged as a marker that is specific to generalized bacterial infections. Moreover, PCT is also closely related to severity of systemic inflammation. Therefore, it can be used to monitor systemic inflammatory course and guide the process of therapeutic decision making. PCT measurement has been used management of severely ill patients due to the fact that its diagnostic capabilities are better than the conventional inflammatory markers.^10^

In the literature PCT levels are increasingly being supported to help improve the capacity to distinguish bacterial from non-bacterial infections.^11^ This can be due to the fact that nonbacterial infections and nonspecific inflammatory reactions are not associated with an increase in PCT levels. In addition, the drop in PCT levels indicates that the patient’s response to antimicrobial therapy is favorable. PCT is therefore a promising marker that makes a rational decision both regarding prescription and the duration of antimicrobial therapy in infected patients.^12^,^13^ In a study, the sensitivity and specificity of procalcitonin in maternal plasma to detect early intra amniotic infection in preterm premature rupture of membrane is 75% and 45%.

As the use of procalcitonin is rapidly increasing for diagnosing the bacterial infections in our general practice, this inflammatory marker may be helpful in early diagnosis of subclinical intra amniotic infection, so we can treat infection early and may prevent maternal and perinatal morbidity and mortality. Therefore, this study was conducted with the aim to determine the diagnostic accuracy of procalcitonin in maternal plasma to detect early intraamniotic infection in PPROM taking high vaginal swab as gold standard.

## METHODS

A cross-sectional study was conducted at the department of Gynaecology and Obstetrics, Liaquat National Hospital (LNH), Karachi, from 1^st^ February 2017 to 31st August 2017. All women with PPROM were included through non-probability consecutive sampling. The sample size was calculated by using WHO calculator. Total 150 women of reproductive age with gestational age 24-37 weeks of singleton pregnancy having preterm premature of membrane were included. Patients with history of previous preterm labour, obvious bacterial infection, condition that may have increased PCT level without bacterial cause, falsely low PCT levels in the presence of bacterial infection were not included in the study.

PPROM was defined as rupture of membranes during pregnancy before 37 weeks gestation. PCT level <0.5 ng/ml was considered as a low-risk of intraamniotic infection and >2.0 ng/ml was considered as a high-risk of bacterial intraamniotic infection. Chorioamnionitis / intraamniotic infection (IAI) was defined as acute inflammation of membranes and placental chorion due to ascending polymicrobial bacterial infection in setting of rupture of membranes. When patient came with PPROM, high vaginal swab was taken with a non-touch technique and was sent for cultures. The culture was considered as positive if bacterial growth colonies growing in high vaginal swab on the basis of lab examination.

Patient demographics and clinical history were taken by the principal investigator. The blood sample was sent to department of microbiology within 30 minutes of collection, sample was then centrifuged to separate the serum to process it on Cobas e411 by ECLIA method. Two controls i.e. PCT1 and PCT2 were run along with the sample for the accuracy of the results. The instrument process the sample within 18 minutes and the results are communicated immediately to the concerned department. Then for confirmation of infection, HVS culture was sent of the same patients irrespective of procalcitonin results whether high or low to see the true positive and false positives.

All the data were compiled and analyzed via statistical package for Social Sciences (SPSS version 23). Frequency and percentage were calculated to estimate the true, positive, true, negative findings. Mean and standard deviation was calculated for age, gestational age, parity and prolactin level. Diagnostic accuracy was measured to determine the sensitivity, specificity, positive predictive value (PPV), negative predictive values (NPV) and the overall diagnostic accuracy of procalcitonin considering HVS culture as gold standard. Stratification with respect to age, gestational age, parity and intra-amniotic infection was done. Post stratification diagnostic accuracy, sensitivity, specificity, PPV and NPV were also calculated.

### Ethical Approval:

(Ref: 0484-2019-LNH-ERC, dated: June 15, 2019)

## RESULTS

Out of total 150 patients, mean age of the patients was 28.78±4.79 years. Mean parity was 1.90±1.38 whereas mean gestational age was 30.79±3.07 weeks ranging from 25 weeks to 36 weeks. Mean procalcitonin level was 0.13±0.24 ng/ml. (Table-I)

**Table-I:** Descriptive statistics of age, parity, gestational age, procalcitonin level (n=150).

	Age (years)	Parity	Gestational Age (Weeks)	Procalcitonin Level (ng/ml)
Mean	28.78	1.90	30.79	0.13
Standard Deviation	4.79	1.38	3.07	0.24
Median	28.00	2.00	31.00	0.03
Range	23	6	11	0.89
Minimum	19	0	25	0.01
Maximum	42	6	36	0.90

Intra-amniotic infection was diagnosed in 48.7% cases through procalcitonin level and 51.3% through HVS CS. The results showed that 67 patients were true positive and 67 patients were true negative. Sensitivity, Specificity, PPV, NPV and accuracy were 87%, 91.8%, 91.78%, 87%, and 89.3% respectively. (Table-II).

**Table-II:** Diagnostic accuracy of procalcitonin in maternal plasma to detect early intra-amniotic infection in PPROM.

Procalcitonin Level	Placental Tissue Hvs Cs		

	Positive	Negative	TOTAL
Positive	67 (True +ve)	6 (False +ve)	73
Negative	10 (False -ve)	67 (True +ve)	77
Total	77	73	150
Sensitivity	87%		
Specificity	91.8%		
Positive predicted value	91.78%		
Negative predicted value	87.0%		
Overall diagnostic accuracy	89.33%		

For females with age ≤30 years, sensitivity, specificity, and diagnostic accuracy were 86.7%, 87.2%, and 86.9% respectively. For females with age >30 years, sensitivity, specificity, and diagnostic accuracy were 88.2%, 100%, and 95.3% respectively. For females with parity ≤2, results showed that sensitivity, specificity, and diagnostic accuracy were 85.5%, 89.8%, and 87.6% respectively. For females with parity >2, sensitivity, specificity, and diagnostic accuracy were 93.3%, 100%, and 96.5% respectively. For females with gestational age ≤32 weeks, sensitivity, specificity, and diagnostic accuracy were 83.9%, 90.4%, and 87.03% respectively. For females with gestational age >32 weeks, sensitivity, specificity, and diagnostic accuracy were 95.2%, 92.5%, and 95.23% respectively. (Table-III).

**Table-III:** Baseline Characteristics and diagnostic accuracy of procalcitonin in maternal plasma to detect early intra-amniotic infection in PPROM (n=150).

	Age ≤30 Years	Age >30 Years	Parity ≤2	Parity >2	Gestational Age ≤32 weeks	Gestational Age >32 weeks
Sensitivity	86.70%	88.20%	87%	87%	87%	87%
Specificity	87.20%	100%	91.80%	91.80%	91.80%	91.80%
PPV	89.60%	100%	91.78%	91.78%	91.78%	91.78%
NPV	83.60%	92.80%	87.00%	87.00%	87.00%	87.00%
Overall diagnostic accuracy	86.90%	95.30%	89.33%	89.33%	89.33%	89.33%

NPV: Negative Predicted Value, PPROM: Preterm premature rupture of the membranes,PPV: Positive Predicted Value.

## DISCUSSION

IAI (intra-amniotic infection) at an early stage is important in females who are at term or in those who have PPROM. In early stages of chorioamnionitis, the signs of infective disease process are usually subtle and are not evident routinely. In the presence of classical clinical signs and symptoms, the patient is said to be suffering from clinical chorioamnionitis / clinical IAI. There is a significant overlap between clinical and histological characteristics of chorioamnionitis. Moreover, subclinical forms of chorioamnionitis may also be evident in the form of preterm labor or in the form of PPROM.^14^ In mothers, chorioamnionitis may result in septic shock, disseminated intravascular coagulation (DIC) and death.^15^,^16^ The maternal serum is an easy way to approach and obtain the material required for diagnosis in a minimally invasive manner. C-reactive protein (CRP), counting of white blood cells, TNF-α, IL-6, IL-10 and G-CSF markers that can be tested from maternal serum. These markers have been used to predict the risk of chorioamnionitis as well as neonatal infection among pregnant females suffering from PPROM. with premature rupture of membranes (PROM).^17^,^18^

PCT has been used as a new parameter to diagnose any generalized / systemic infections. Increment in PCT caused by bacterial infections correlates well with severity of disease as well as mortality.^19^ In the field of obstetrics, very limited data is available regarding PCT, especially local data is very deficient, this study is useful addition in local studies as it shows significant accuracy in the diagnosis of intra-amniotic infections. PCT levels can be detected in plasma almost two hours after endotoxins injection. The levels rise within six to eight hours and reach a plateau after 20 to 72 hours. It normalizes within two to three days.^19^,^20^ Procalcitonin is superior than other parameters like WBC & CRP but in some studies still CRP found more reliable.^21^,^22^

In this study we determined the diagnostic accuracy of procalcitonin in maternal plasma for detection of early intraamniotic infection in PPROM taking high vaginal swab as gold standard. The study included 150 patients having gestational age of 24-37 weeks diagnosed as having PPROM. The results of our study show that PCT has a high sensitivity of 87% in diagnosis of early intraamniotic infection. The reported sensitivity is higher than the one reported by Torbe et al. Another study reported the sensitivity of PCT of 50% in diagnosis of chorioamnionitis.^23^ A study conducted by Oludag et al, reported a slightly higher sensitivity of 92.3% for subclinical IAI as compared to our study.^12^

Our study results have shown that PCT has a high specificity of 91.8% in diagnosis of early intraamniotic infection. This reported sensitivity is higher as compared to the ones reported in literature.^10^, ^19^, ^20^ Moreover, according to literature, PCT also has a higher sensitivity and specificity than CRP for diagnosis of chorioamnionitis.^24^

Many changes occur in the inflammatory system, the cortisol and other factors during gestation, which normally increase the WBC count and peak at the end of gestation. (25) Therefore, the levels of WBCs may not accurately depict clinical conditions present in pregnant females.

### Limitations of the stud: .

This was a single institute study. Moreover, we did not compare the PCT levels with other inflammatory markers such as WBC count and CRP levels. Strengths of our study include prospective data collection from all of its participants. It is recommended that further multicenteric studies on larger sample size shall be carried out to provide better understanding to the current topic.

## CONCLUSION

The detection of early intra-amniotic PPROM infection was found to be satisfactory for the diagnosis of mother’s blood procalcitonin levels. The maternal blood procalcitonin level is therefore a clinically useful biomarker, non-invasive and reliable for detecting early intra-amniotic infection in PPROM cases.

### Authors Contribution:

**QAS & RF:** Conceived, designed and did editing of manuscript and are responsible for integrity of the study.

**QAS & RF:** Did data collection, statistical analysis and manuscript writing.

**QAS & RF:** Did review and final approval of manuscript.

**RF:** Did data collection.
